# Energy Balance Related Behaviour: Personal, Home- and Friend-Related Factors among Schoolchildren in Europe Studied in the ENERGY-Project

**DOI:** 10.1371/journal.pone.0111775

**Published:** 2014-11-05

**Authors:** Saskia J. te Velde, Amika Singh, Mai Chinapaw, Ilse De Bourdeaudhuij, Natasa Jan, Eva Kovacs, Elling Bere, Froydis N. Vik, Bettina Bringolf-Isler, Yannis Manios, Luis Moreno, Johannes Brug

**Affiliations:** 1 EMGO Institute for Health and Care Research and the Department of Epidemiology & Biostatistics, VU University Medical Center, Amsterdam, the Netherlands; 2 EMGO Institute for Health and Care Research and the Department of Occupational and Public Health, VU University Medical Center, Amsterdam, the Netherlands; 3 Ghent University, Department of Movement and Sport Sciences, Ghent, Belgium; 4 Slovenian Heart Foundation, Ljubljana, Slovenia; 5 Eva Kovacs, Department of Paediatrics, Pecs University, Pecs, Hungary; 6 Department of Public Health, Sport and Nutrition, University of Agder, Kristiansand, Norway; 7 Swiss Tropical and Public Health Institute, Basel, Switzerland, and University of Basel, Basel, Switzerland; 8 Department of Nutrition and Dietetics, Harokopio University, Athens, Greece; 9 GENUD (Growth, Exercise, Nutrition and Development) Research Group, Universidad de Zaragoza, Zaragoza, Spain; The University of Kansas Medical Center, United States of America

## Abstract

**Objective:**

To design interventions that target energy balance-related behaviours, knowledge of primary schoolchildren's perceptions regarding soft drink intake, fruit juice intake, breakfast consumption, TV viewing and physical activity (PA) is essential. The current study describes personal beliefs and attitudes, home- and friend-related variables regarding these behaviours across Europe.

**Design:**

Cross-sectional study in which personal, family and friend -related variables were assessed by validated questionnaires, and dichotomized as favourable versus unfavourable answers. Logistic regression analyses were conducted to estimate proportions of children giving unfavourable answers and test between-country differences.

**Setting:**

A survey in eight European countries.

**Subjects:**

A total of 7903 10–12 year old primary schoolchildren.

**Results:**

A majority of the children reported unfavourable attitudes, preferences and subjective norms regarding soft drink, fruit juice intake and TV viewing accompanied with high availability and accessibility at home. Few children reported unfavourable attitudes and preferences regarding breakfast consumption and PA. Many children reported unfavourable health beliefs regarding breakfast consumption and TV viewing. Substantial differences between countries were observed, especially for variables regarding soft drink intake, breakfast consumption and TV viewing.

**Conclusion:**

The surveyed children demonstrated favourable attitudes to some healthy behaviours (PA, breakfast intake) as well as to some unhealthy behaviours (soft drink consumption, TV viewing). Additionally, many children across Europe have personal beliefs and are exposed to social environments that are not supportive to engagement in healthy behaviours. Moreover, the large differences in personal, family and friend-related variables across Europe argue for implementing different strategies in the different European countries.

## Introduction

According to estimates of the International Obesity Task Force [1] up to 200 million school-aged children are currently either overweight or obese. Recent data from the European Commission funded ENERGY-project [2] showed that more than 20% of the participating 10–12-year-old children from 7 European countries were overweight varying from 14% to 44% depending on the country and sex [2]. Some specific energy balance-related behaviours (EBRB) are regarded as particularly important with respect to weight status, such as sport participation, TV viewing, the consumption of sugar containing beverages and breakfast skipping [3]–[5]. There may be a specific role for sugar containing beverages in the development of overweight based on the parallel increase of sugared beverage consumption and overweight [6]. Also, fruit juices have similar energy densities as sugar-sweetened beverages and may as well contribute to excessive weight gain [7], [8]. However, people may perceive fruit juices as healthy and do not recognize the excess energy of high fruit juice intake. Similarly, skipping breakfast may be seen as an effective strategy to lose weight or prevent weight gain, and therefore be perceived as an effective weight management behaviour [9], while evidence indicates that skipping breakfast is inversely associated with overweight and obesity. Despite previous observations that most children and adolescents are aware of the health benefits of physical activity and sport participation, many do not meet the physical activity recommendations [10], which may be due to unfavourable home-related variables [11], [12]. For the development of future interventions it is therefore essential to study and compare personal perceptions, beliefs and attitudes of schoolchildren regarding soft drink fruit juice intake and daily breakfast consumption. Moreover, such descriptive information is important input for public health professionals. Factors in the home environment are important for shaping and establishing energy balance-related behaviour of school-aged children [13]–[15]. Higher availability and accessibility of unhealthy food items are related to higher intake [16]–[20], whereas having family rules regarding intake of sugar containing beverages is associated with lower intake [15], [20], [21]. In the same line, adolescent sport participation has been positively associated with availability of sport equipment and parental rule setting [22].

From our recent observations based on data gathered within the ENERGY-project [2], we learned that engagement in specific EBRBs differ largely between the participating countries [2]. Based on these observations we expect personal, home- and friend-related variables associated with these behaviours to also vary between countries. Therefore, the current study aims to describe the self-reported scores, i.e. perceptions, on personal beliefs and attitudes, home- and friend-related variables related to intake of soft drinks and fruit juices, breakfast consumption, physical activity and TV viewing in eight European countries.

## Methods

### Ethics

The ENERGY-project adheres to the Helsinki Declaration and the conventions of the Council of Europe on human rights and biomedicine. All participating countries obtained ethical clearance from the relevant ethical committees and ministries; in Belgium the survey was approved by the Medical Ethics Committee of the University Hospital Ghent; in Greece the survey was approved by the Bioethics Committee of Harokopio University; in Hungary the survey was approved by the Scientific and Ethics Committee of Health Sciences Council; in the Netherlands the survey was approved by the Medical Ethics Committee of the VU University medical center; in Norway the survey was approved by the National Committees for Research Ethics in Norway; in Slovenia the survey was approved by the National Medical Ethics Committee of the Republic of Slovenia; in Spain the survey was approved by Clinical Research Ethics Committee of the Government of Aragón, and in Switzerland the survey was approved by the ethic committees of Basel, St. Gallen, Bern and Aargau. Furthermore, research permission was, if necessary, obtained from local school authorities (local school boards and/or headmasters). All students and their parents received written information on the project prior to enrolment in the study. Completion of the questionnaires was voluntary.

A description of the rationale and organization of the ENERGY-project [23] and a comprehensive description of the design, procedures, and methodology of the ENERGY school-based survey [24] are published elsewhere. The data collection manual and survey questionnaires for the ENERGY cross-sectional survey are available online at http://www.projectenergy.eu.

### Sample and procedure

Seven countries from the ENERGY consortium, Belgium, Greece, Hungary, the Netherlands, Norway, Slovenia and Spain participated in the cross-sectional survey. An eighth country, Switzerland, joined in a later phase [25]. Each country was represented by a local research institute, with each partner being responsible for the data collection in their country. The standardized procedure for sampling, data collection, and data handling for the survey was the same in all countries [24].

The cross-sectional survey was carried out in primary schools among 10–12 year old children. The recruitment and data collection took place from March-July 2010 (Belgium, Greece, Hungary, the Netherlands, Norway, Slovenia and Spain) and between June and December 2010 (Switzerland). Sampling was nationally representative in Greece, Hungary, the Netherlands, and Slovenia. In Spain, schools in the region of Aragón were selected, Belgium selected schools from Flanders (i.e. the northern Dutch-speaking part of Belgium), Norway selected schools from the southern regions of the country and in Switzerland children from the German-speaking part of Switzerland were included [25]. Recruitment methods and response rates are described in more detail elsewhere [24]. Briefly, between 15 (Slovenia) and 37 (Greece) schools participated, with a wide range in response rates at the school level (5% in the Netherlands – 100% in Slovenia). Response rates at the child level were in general high (>80%), but in Hungary (33%), Norway (45%) and Spain (43%) lower response rates were obtained, mainly because of parents not returning completed parental consent forms.

Children completed the child questionnaire during one school hour in the presence of a research assistant or project worker who guided the completion of the questionnaire according to a standardized protocol. The children brought home parental questionnaire to be completed by one of the parents. In total, 7915 children completed the questionnaires of whom 99 did not provide data on variables related to the energy balance behaviours and were therefore excluded from the analyses. The response rate among parents was much lower. For the current study data from the parent questionnaire was available for a maximum of 6773 (86%) children, depending on the variable.

### ENERGY-child questionnaire

The ENERGY-child questionnaire was developed in order to assess EBRB of the child as well as personal, family and friend-environmental determinants related to these EBRB. The questionnaire was divided into eight sections, i.e. (A) Demographic characteristics; (B) Soft drinks and spending pocket money on soft drinks; (C) Fruit juices; (D) Breakfast behaviour; (E) Physical activity behaviour; (F) Screen viewing behaviour; and (G) Dieting behaviour. A reliability and validity study was conducted in which children completed the questionnaire twice, with one week in-between the two measurements (n = 730 in the test-retest reliability study; n = 96 in the construct validity study). Construct validity was evaluated by means of a cognitive interview. Results demonstrated that the ENERGY-child questionnaire, assessing EBRB of the child as well as personal, family, and school-environmental determinants related to these EBRB, has a test-retest reliability that was good (Intra class correlation coefficient (ICC) between 0.6–0.8) to excellent (ICC >0.80) in 115 (76.6%) items and moderate (ICC between 0.4–0.6) in 34 (22.7%) items. Construct validity appeared to be good to excellent for 70 out of 150 items (46.7%), as indicated by ICCs >.60 or percentage agreement ≥75%. For the remaining part, the ICCs of 39 items (26.0%) indicated moderate construct validity and 41 items (27.3%) indicated poor construct validity, which included the assessment of attitude and parental norms [26].

### Variables related to EBRB

We assessed the personal, family and school environments variables with single questionnaire items. Existing behavioural models such as the Theory of Planned Behaviour (TPB) [27], Socio-Ecological Models [28], and the Environmental Research framework for weight Gain prevention (EnRG-framework) [29] describe direct and mediated pathways between behavioural determinants, including personal and environmental variables, and (intentions to perform) the behaviours. The ENERGY Cross-sectional study aimed to assess a broad range of these presumed determinants taking different theories and health behaviour models into account, in line with the approach advocated in Intervention Mapping [30]. Based on the TPB we included attitude, subjective norm and perceived behavioural that are all linked to behaviour (through intentions); based on habit-theory [31] we included automaticity, which describes a less conscious and less rationale decision-making process for engaging in the behaviour; based on existing literature we further included knowledge and health beliefs. Based on socio-ecological models and the EnRG-framework we included environmental variables such as perceived modelling and parenting practices (rules) that are presumed to have both a direct link with the behaviour as well as an indirect link through the cognitive variables (e.g. attitude, perceived behavioural control). Exact formulations of the questions will presented in the tables displaying the findings. A test-retest reliability study, conducted among children from six countries, showed good to excellent test-retest reliability as indicated by ICCs >.60 or percentage agreement ≥75% for most questions (see [26] for more details). The construct validity was estimated by comparing the answers from the questionnaires with results from a cognitive interview in a small sample of children (n = 96) [26]. Results indicated that this construct validity was moderate to good for most questions related to soft drink and fruit juice intake, but poor to moderate construct validity was observed for most variables related to breakfast consumption, physical activity and TV viewing, which is most likely due to the lack of a gold standard for estimating the validity of cognitions related to EBRB (see [26] for more details).

Briefly, regarding the consumption of *soft drinks* we assessed the children's attitudes, taste preferences (liking), automaticity, perceived behavioural control for NOT drinking soft drinks, health belief with respect to soft drinks contributing to getting overweight, perceived parental norm and modelling, to what extent parents allowed soft drink intake in general (‘parental allowance’) and if asked for (‘accessibility’), perceived family rules, whether products were bought on request, home availability, perceived friend's norm and perceived friend's modelling.

Regarding the intake of *fruit juices* we assessed children's attitude, their knowledge of the recommendation, health belief regarding fruit juice and overweight, perceived parental allowance, perceived family rules and perceived home availability.

Regarding *breakfast consumption* we assessed children's attitude, taste preferences, automaticity, perceived behavioural control, health beliefs with respect to unnecessary weight gain regarding eating and NOT eating breakfast, perceived parental norm and modelling, if breakfast was eaten together with parents (‘perceived co-participation’), perceived parental encouragement, family rules and home availability, and if products were bought on children's request.

Regarding *physical activity/sports* we assessed the children's attitude, knowledge of the recommendation (i.e. at least 60 minutes a day), health belief regarding physical activity/sport and overweight, liking of physical activity/sport, automaticity, perceived behavioural control, parental norm and modelling, perceived parental encouragement, perceived parental support, family rules, parental general allowance, parental allowance of a favourite physical activity/sport, parental co-participation, perceived friend's norm and perceived friend's modelling.

Regarding *TV watching* we assessed the children's attitude, knowledge of the recommendation (i.e. a maximum of 2 hours a day), health beliefs regarding TV watching and overweight, liking, automaticity, perceived behavioural control for NOT watching TV, perceived parental norm, perceived parental modelling, availability of a TV in the bedroom, family rules, general allowance, allowance on request, parental co-participation, perceived friend's norm and perceived friend's modelling.

All variables, except questions about family rules and availability of a TV in the bedroom, were assessed on 5-point scales (−2 to +2). As most variables showed strongly skewed distributions, all variables were dichotomized so that unfavourable categories (e.g. combining the two upper or two lower categories) could be presented (the Tables provide information on which answer categories were combined). For the variables assessing children's knowledge about recommendations we combined incorrect answers, or answers for which there was consensus that they are incorrect. As there are no clear guidelines for fruit juice intake, or guidelines vary by country, we combined the answer categories *‘not to drink FJ at all’, ‘drink as much FJ as you want’ and ‘don’t know’* representing the unfavourable response. We coded ‘*drink not more than 1 glass a day’* as the favourable response.

#### Demographics

Parents reported their own level of education, as well as the level of education of the other parent/caregiver and the countries of birth of themselves, their partner and their child. Parental education was categorized as being high (i.e at least one parent with more than 14 years of education) or low (i.e. both parents less than 14 years of education), roughly distinguishing families with at least one caregiver who has completed medium or higher vocational, college or university training from other families [32]. A dichotomous variable was created to distinguish parents with a ‘native’ background (i.e., both parents were born in the country of administration) from those with a ‘non-native’ background (i.e., at least one parent was born in another country). Children reported their sex and birth dates (i.e. month and year of birth).

### Statistical analyses

Proportions were calculated for the whole sample and for each country separately. Due to the large number of variables analysed in the current study, the findings are only mentioned in the results sections if observations substantially vary between countries, i.e. ±10 percent points from the total sample prevalence rate.

Logistic regression analyses were used to estimate proportions adjusted for age, sex and parental education. Countries were compared by means of logistic regression analyses (using dummy coding, and varying the reference category) and rank ordered from most favourable to less favourable. Countries with a different rank differed statistically significantly from each other.

As the ENERGY cross-sectional survey applied a nested design, with children nested in schools, proxies for intra class correlation coefficients (ICC) were calculated as suggested by Twisk [33]. All ICCs were considered low (all <0.08, except for friends norm regarding fizzy drinks, ICC  = 0.108). We therefore did not adjust for the nested design.

Country-specific results and differences between countries are only discussed if the country specific proportion differed 10 or more percent points from the (predicted) proportion of the whole sample and/or when there is a wide variation between countries as indicated by more than 10 percent point differences.

## Results

The mean age of the total sample was 11.6 (±0.75) years, but this differed slightly between countries, and 52% were girls (see Table 1). As shown in previous publications about this sample [2], [34] 17.3% had a non-native background. The latter proportion was much higher in Switzerland (36%) and Greece (31%). In the total sample, 65% of the parents had at least 14 years of education, which differed by country ranging from 40% in Switzerland to 84% in Belgium (see Table 1, and see previous publications about this sample, e.g.: [34]–[37]).

**Table 1:** Sample characteristics.

			Belgium	Greece	Hungary	the Netherlands	Norway	Slovenia	Spain	Switzerland	Total	p^2^
	N		996	1085	1022	919	1000	1176	1022	596	7816	
Sex	Girl	%	52.1	53.9	55.1	50.4	51.9	51.2	51.8	47.8	52.0	0.139
Age		Mean	11.5	11.3	12.2	11.8	12.0	11.4	11.4	11.6	11.6	<0.001
		SD	.68	.63	.64	.76	.74	.62	.64	.80	.75	
Ethnic background	N		745	969	922	392	840	1008	956	550	6382	
	Non-native	%	8.3	31.1	4.7	15.3	15.6	20.2	10.9	36.0	17.3	<0.001
Parental educational level	N		666	891	763	349	718	897	880	546	5710	
	at least one parent > = 14 years of education	%	84.2	51.7	58.1	77.7	74.5	56.3	80.6	40.3^1^	64.9	<0.001

N – total number with available data on this variable.

1 In Switzerland the first two years of education are generally not referred to as ‘school’ education. Therefore the proportion of higher educated people in Switzerland is probably an underestimation;

2 p-value for between country differences, tested with χ^2^ in case of dichotomous variables and with ANOVA in case of a continuous variable (age).

### Soft drink intake

The descriptive results of the variables regarding soft drink intake and the between-country comparisons are presented in Table 2. Adjustment for age and sex did not substantially influence the estimates (i.e. ≤1 percent point). Adjusting for parental education did only marginally affect the estimated proportions for the correlates related to soft drink intake (i.e. change ≤2 percent points). Therefore, and because of the reduced samples size after adjustment for parental education, we solely present the observed unadjusted values and mention those exceptions (between brackets) where adjustment for parental education influenced the estimate with ≥3 percent points.

**Table 2:** Observed (%) and ranks^1^ for the variables related to **soft drink** consumption for the total sample and by country.

Question or statement (label or construct)	Answer categories^2^		Belgium	Greece	Hungary	Netherlands	Norway	Slovenia	Spain	Switzerland	Total
**Personal**											
I think that drinking soft drink is …(attitude)	*Good; very good*	%	23	**8**	**53**	18	**9**	30	13	24	22
		rank	5	1	6	4	1	5	3	4	
I like the taste of soft drink …(liking)	*agree a bit; agree*	%	**88**	**53**	82	**92**	**91**	**59**	74	72	76
		rank	5	1	4	6	5, 6	2	3	3	
Drinking soft drink is something I do without even thinking (automaticity)	*agree a bit; agree*	%	**47**	22	33	**49**	32	33	**13**	38	33 (30)
		rank	5	2	3	5	3	3, 4	1	4	
I find NOT drinking soft drink …(perceived behavioural control)	*Difficult; very difficult*	%	18	11	13	19	8	5	9	15	12
		rank	5	3, 4	4	5	2	1	2, 3	4	
I think that drinking soft drink will make me fat. (health belief)	*disagree a bit; - fully disagree*	%	18	**7**	13	27	22	27	14	**33**	19
		rank	3	1	2	4	3	4	2	5	
**Family environment**											
If I drink soft drinks my parents think this is. (parental norm)	*Good; –very good*	%	17	8	18	**36**	**2**	12	9	4	13
		rank	5	3	5	6	1	4	3	2	
How often do your parents drink soft drinks.. (parental modelling)	*Often; always*	%	24	15	**33**	19	11	11	14	7	17
		rank	5	3	6	4	2	2	3	1	
If I ask my parents for a drink fizzy drink, I get one. (accessibility)	*Often; always*	%	41	22	**50**	**46**	**11**	27	**17**	21	29
		rank	5	3	6	5, 6	1	4	2	2, 3	
I am allowed to take drink soft drinks whenever I want. (allowance)	*Often; always*	%	33	**15**	**43**	**41**	**12**	34	13	31	27
		rank	3, 4	2	4, 5	5	1	4, 5	1,2	3	
Do your parents/care givers have rules about how many soft drink you are allowed to drink? (rules)	*No*	%	51	**33**	56	**60** (56)^3^	42	42	35	**70**	47
		rank	3	1	3	4	2	2	1	5	
If you ask your parent to buy certain soft drinks, they will buy it. (bought on requested)	*Often; always*	%	39	21	**43**	31	25	27	23	20	29
		rank	4	1, 2	4	3	2	3	2	1	
Are there usually soft drinks at your home? (availability)	*Often; always*	%	**67**	28	**56**	**77**	44	**27**	40	36	46
		rank	5	1	4	6	3	1	3	2	
**Friend environment**											
If I drink soft drinks my friends think this is..... (friend norm)	*Good; very good*	%	50	**28**	**79**	**59**	**13**	49	49	31	45
		rank	3	2	5	4	1	3	3	2	
How often do your friends drink soft drinks? (friend modelling)	*Often; always*	%	52	42	**78**	**57**	**23**	44	**32**	41	46
		rank	4	3	5	4	1	3	2	3	

**Bold –** proportions that differ ≥10 percent points from the proportion observed in the total sample.

1 rank orders are based on between-country comparisons by logistic regression analyses and shown in the order of most favourable to least favourable. Countries with the same rank do not differ from each other (p>0.05).

2 potential categories are ‘fully disagree’, disagree a bit’, ‘neither disagree nor agree’, ‘agree a bit’, ‘fully agree’ or ‘always’, ‘often’, ‘sometimes’, ‘not often’, ‘never’ or ‘very good’, ‘good’, ‘neither good nor bad’, ‘bad’, ‘very bad’.

3 between brackets the estimated proportion after further adjustment for parental educational level if this differed ≥3 percent points from the unadjusted proportion.

#### Personal variables of soft drink intake

For most variables the observed proportions varied between countries (Table 2). In general Greece, Norway and Spain showed the most favourable pattern regarding the personal factors, while Belgium and the Netherlands showed the most unfavourable pattern with regard to soft drink intake.

#### Family-environmental variables of soft drink intake

In general soft drinks were perceived as available and accessible for many of the participating children. However, all family-related variables varied widely between the countries (see Table 2). In general, Belgium, Hungary and the Netherlands showed more unfavourable patterns compared to the other countries.

#### Friend- environmental variables of soft drink intake

Overall, 45% of the children reported unfavourable attitudes and 46% reported unfavourable friend norms regarding soft drink intake. These proportions varied between countries (see Table 2) with Hungarian children showing the most unfavourable and the Norwegian children showing more favourable friend norms and modelling.

### Fruit juice intake

The descriptive results regarding the correlates of fruit juice intake and between-country comparisons are presented in Table 3. We solely present the observed unadjusted values, because adjustment for age, sex and parental education did not substantially influence the estimates (i.e. change ≤2 percent points).

**Table 3:** Observed (%) and ranks^1^ for the variables related to **fruit juice (FJ)** consumption for the total sample and by country.

Question or statement (label or construct)	Answer categories^2^		Belgium	Greece	Hungary	Netherlands	Norway	Slovenia	Spain	Switzerland	Total
**Personal**											
I think that drinking FJ is …(attitude)	*Good; very good*	%	61	**22**	51	**71 (75)** 3	**78**	62	48	61	56
		rank	3	1	2	4	5	3	2	3	
I think that it is recommended to.....(knowledge)	*drink as much as you like; don't know*	%	72	68	63	**80**	54	53	54	68	63
		rank	3	3	2	4	1	1	1	3	
I think that drinking FJ will make me fat.. (health belief)	*disagree a bit; fully disagree*	%	68	71	**48**	69	65	61	60	73	64
		rank	4, 5	5	1	4, 5	3, 4	2, 3	2	5	
**Family environment**											
I am allowed to take drink FJ whenever I want..... (allowance)	*Often; always*	%	72	67	58	**75 (68)** 3	55	66	59	63	64
		rank	4	3	1	4	1	3	2	2	
Do your parents/care givers have rules about how many FJ you are allowed to drink? (rules)	*No*	%	81	**60**	78	**86**	69	66	63	**90**	73
		rank	4	1	3	5	2	2	1, 2	6	
Are there usually FJ at your home..... (availability)	*Sometimes; always*	%	68	73	58	72	57	63	74	57	66
		rank	3	4, 5	1	3, 4	1	2	5	1	

**Bold** – indicates that the observed proportion differs 10 or more percent points from the proportion in the Total sample.

1 rank orders are based on between-country comparisons by logistic regression and shown in the order of most favourable to least favourable. Countries with the same rank do not differ from each other (p>0.05).

2 potential categories are ‘fully disagree’, disagree a bit’, ‘neither disagree nor agree’, ‘agree a bit’, ‘fully agree’ or ‘always’, ‘often’, ‘sometimes’, ‘not often’, ‘never’ or ‘very good’, ‘good’, ‘neither good nor bad’, ‘bad’, ‘very bad’.

3 between brackets the estimated proportion after further adjustment for parental educational level if this differed ≥3 percent points from the unadjusted proportion.

#### Personal correlates of fruit juice intake

Overall, most children had an unfavourable attitude, did not know the correct recommendation and reported an unfavourable health belief with regard to fruit juice intake. However, proportions varied between countries, especially for the unfavourable attitude (see Table 3). In general, children in Spain and Greece showed more favourable personal factors, while Dutch children showed more unfavourable personal factors.

#### Family-environmental correlates of fruit juice intake

Overall, fruit juices were available and accessible for the majority of the children (Table 3). Not having family rules varied strongly between the countries. In general, Norwegian children reported more favourable family factors regarding fruit juice intake, while Dutch and Belgian children reported a less favourable pattern.

### Breakfast consumption

The descriptive results regarding the variables related to breakfast consumption and between-country comparisons are presented in Table 4. We solely present the observed unadjusted values, because adjustment for age and sex did not substantially influence the estimates (i.e. change ≤2 percent points). Further adjustment for parental education did only affected two estimates (preferences and perceived behavioural control) in the Dutch sample (≥3 percent points change), which are presented in the tables between brackets.

**Table 4:** Observed proportions (%)and ranks^1^ for the variables related to **breakfast consumption (BF)** consumption for the total sample and by country.

Question or statement (label or construct)	Answer categories^2^		Belgium	Greece	Hungary	Netherlands	Norway	Slovenia	Spain	Switzerland	Total
**Personal**											
I think that eating BF is … (attitude)	*Bad;- very bad*	%	0.4	1.0	1.8	0.3	0.6	1.4	0.2	0.2	0.8
		rank	1	2	2	1	1, 2	2	1	1, 2	
I think it is recommended to.... (knowledge)	*to skip breakfast - eat BF on schooldays; don’t know*	%	20	35	34	23	**9**	34	13	**43**	26
		rank	3	4	4	3	1	4	2	5	
I like eating BF..... (preferences)	*disagree a bit; - fully disagree*	%	3	5	8	4 (1)^3^	4	8	4	3	5
		Rank	1	2	2, 3	1	1	3	1, 2	1	
Eating BF is something I do without even thinking..... (automaticity)	*disagree a bit; fully disagree*	%	19	27	25	**16**	19	35	**49**	18	27
		Rank	1, 2	3	3	1	2	4	5	1, 2	
I find eating BF every day..... (perceived behavioural control)	*Difficult; very difficult*	%	7	10	8	5 (2)^3^	4	7	3	6	6
		Rank	4	5	4	2, 3	1, 2	4	1	3, 4	
I think that NOT eating BF will make me fat..... (health belief)	*disagree a bit; fully disagree*	%	**45**	60	67	**45**	62	**77**	71	**74**	62
		Rank	1	2	3	1	2	5	3, 4	4, 5	
I think that eating BF will make me fat..... (health belief)	*agree a bit; fully agree*	%	35	38	44	34	**22**	**22**	51	31	35
		Rank	2, 3	3	4	2, 3	1	1	4	2	
**Family environment**											
If I eat BF my parents think this is..... (parental norm	*Bad'; very bad*	%	0.1	0.6	0.5	0.1	0.4	1.6	0.2	0.3	0.5
		Rank	1	1	1	1	1	2	1	1	
How often do your parents eat BF? (parental modelling)	*not often; never*	%	6	15	7	1	2	13	1	7	7
		rank	2	3	2	1	1	3	1	2	
My parents encourage me to eat BF every day.....(parental encouragement)	*disagree a bit; fully disagree*	%	21	12	**4**	**34**	18	13	21	21	18
		rank	3	2	1	4	3	2	3	3	
Do your parents/care givers have rules about whether you should eat breakfast? (rules)	*No*	%	62	59	65	54	**50**	64	**50**	**81**	60
		rank	2, 3	2	3	1	1	3	1	4	
If you ask your parents to buy certain BF products, they will buy it..... (bought on request)	*Often; always*	%	**61**	**37**	**64**	54	47	56	**36**	**36**	50
		rank	4	1	4	3	2	3	1	1	
Are there usually BF products at your home? (availability)	*not often; never*	%	1	3	2	1	1	4	1	1	2
		rank	1	3	2, 3	1, 2	1	3	1, 2	1, 2	
How often do you eat BF with your parents.? (co-participation)	*Never; once a week*	%	**24**	**52**	**44**	23	27	36	33	25	34
		rank	1	4	3	1	1	2	2	1	
**Friend environment**											
If I eat BF my friends think this is..... (friend norm)	*Bad; very bad*	%	.2	1.3	2.5	.3	.2	1.9	.6	.5	1.0
		rank	1	2	2	1	1	2	1	1	
How often do your friends eat BF? (friend modelling)	*not often; never*	%	2	5	4	2	1	8	1	4	3
		rank	2, 3	4	3, 4	2	1	5	1, 2	4	

**Bold** – indicates that the observed proportion differs 10 or more percent points from the proportion in the Total sample.

1 rank orders are based on between-country comparisons by logistic regression and shown in the order of most favourable to least favourable. Countries with the same rank do not differ from each other (p>0.05).

2 potential categories are ‘fully disagree’, disagree a bit’, ‘neither disagree nor agree’, ‘agree a bit’, ‘fully agree’ or ‘always’, ‘often’, ‘sometimes’, ‘not often’, ‘never’ or ‘very good’, ‘good’, ‘neither good nor bad’, ‘bad’, ‘very bad’.

3 between brackets the estimated proportion after further adjustment for parental educational level if this differed ≥3 percent points from the unadjusted proportion.

#### Personal correlates of breakfast consumption

Overall, children showed favourable patterns regarding attitude and taste preferences for breakfast consumption, but unfavourable patterns regarding health beliefs (Table 4). Incorrect knowledge of the recommendation, low automaticity and incorrect health beliefs varied substantially between the countries.

In general, children living in the Netherlands, Belgium and Norway showed a more favourable pattern regarding the personal variables of breakfast consumption, while children from Hungary and Slovenia showed a less favourable pattern.

#### Family-environmental variables of breakfast consumption

Overall, few children reported unfavourable parental norms and modelling, and low availability of breakfast products. Sixty percent of the children reported that no rules were in place. Not having rules, low parental encouragement, frequent buying on request and low parental co-participation varied substantially between countries. In general, a somewhat more unfavourable pattern was observed in Switzerland and a somewhat less unfavourable pattern in Norway.

#### Friend environmental variables of breakfast consumption

Overall, less than 3% and 8% of the children reported unfavourable friend norms and friend modelling respectively, which was consistent across the countries.

### Physical activity and sport participation

The descriptive results regarding the variables related to physical activity and sport participation and between-country comparisons are presented in Table 5. We solely present the observed unadjusted values, because adjustment for age and sex did not substantially influence the estimates (i.e. change ≤2 percent points). Further adjustment for parental education did only affect some estimates in the Dutch, Norwegian and Slovenian samples (≥3 percent points change). These adjusted estimates are presented between brackets in Table 5.

**Table 5:** Observed proportions (%) and ranks^1^ for the variables related to **physical activity (PA)/sport** for the total sample and by country.

Question or statement (label or construct)	Answer categories^2^		Belgium	Greece	Hungary	Netherlands	Norway	Slovenia	Spain	Switzerland	Total
**Personal**											
I think that PA/sports is…(attitude)	*Bad; very bad*	%	0	0	0	0	0	0	0	0	0
		rank	1	1	1	1	1	1	1	1	
I think it is recommended for children my age… (knowledge)	*Less than 1 hour; day or don’t know*	%	66	55	59	64 (67)^3^	54	46 (43)^3^	58	67	58
		Rank	4	2, 3	3	4	2	1	2, 3	4	
I think NOT doing PA/sports will make me fat (health belief)	*disagree a bit; fully disagree*	%	13	18	10	9	10	19	16	13	14
		Rank	2, 3	4	2	1	1, 2	4	3, 4	2	
I like doing PA/sports (preference)	*disagree a bit; fully disagree*	%	0	0	2	1	2	2	1	1	1
		Rank	1	1	3	1, 2	2, 3	3	1	1, 2	
Doing PA/sports is something that I do without even really thinking about (automaticity)	*disagree a bit; fully disagree*	%	19	23	22	19	20	27	**35**	**13**	23
		Rank	4	3, 4	2, 3	4	4	2	1	5	4
I find doing PA/sports for 1 hour everyday… (perceived behavioural control)	*Difficult; very difficult*	%	3	4	4	2	3	2	2	2	3
		Rank	1, 2	1	1, 2	3	2, 3	2, 3	3	2, 3	
**Family environment**											
If I do PA/sports, my parents/care givers think this is (parental norm)	*Bad; very bad*	%	0	1	1	0	1	2	0	0	1
		Rank	1, 2	1	1	1	1	2	1	1	
How often do your parents/care givers do PA/sports? (parental modelling)	*not often; - never*	%	15	**51**	17	**8** (5)^3^	**11**	21	28	19	22
		rank	5	1	4, 5	7	6	3	2	3, 4	
My parents/care givers encourage me to do PA/sports (parental encouragement)	*disagree a bit; fully disagree*	%	9	7	**2**	**15**	8	**4**	8	**12**	8
		rank	4	2, 3	1	6	4, 5	1, 2	3, 4	5	
My parents/care givers help me if I need something for my sports (parental support)	*disagree a bit; fully disagree*	%	2	3	2	3	2	2	2	2	2
		Rank	1, 2	2	1, 2	1, 2	1, 2	1, 2	1	1, 2	
Do your parents/care givers have rules about whether you should be physically active/do sports? (rules)	*No*	%	71	**52**	75	73 (70)^3^	65	60	61	**84**	67
		Rank	4	1	4	4	3	2	2, 3	5	
Do your parents/care givers allow you to take part in physical activity/do sports? (allowance)	*No*	%	3	3	2	4	1	2	2	1	2
		Rank	4	3, 4	2, 3	4	1	2, 3	2	1, 2	
If you indicate that you like a certain physical activity/sports will your parents/care givers allow you to do it? (specific allowance)	*not often; never*	%	1	4	1	2	2	3	3	2	2
		Rank	1	3	1, 2	1, 2, 3	2, 3	2, 3	2, 3	1	
How often do you take part in physical activity/do sports with your parents care givers? (co-participation)	*Never; less than once a week*	%	77	74	64	72	81	**57**	**58**	**89**	70
		Rank	3, 4	2 3	1	2	4	1	1	5	
**Friend environment**											
If I do PA/sports, my friends think this is… (friend norm)	*Bad; very bad*	%	0	0	1	0	0	1	0	0	0
		Rank	1, 2	1, 2	2	1, 2	1, 2	1, 2	1	1, 2	
How often do your friends do PA/sports? (friend modelling)	*not often; never*	%	1	5	4	1	2	4	2	1	2
		rank	1	3	3	1, 2	2	3	2	1, 2	

**Bold** – indicates that the observed proportion differs 10 or more percent points from the proportion in the Total sample.

1 rank orders are based on between-country comparisons by logistic regression and shown in the order of most favourable to least favourable. Countries with the same rank do not differ from each other (p>0.05);

2 potential categories are ‘fully disagree’, disagree a bit’, ‘neither disagree nor agree’, ‘agree a bit’, ‘fully agree’ or ‘always’, ‘often’, ‘sometimes’, ‘not often’, ‘never’ or ‘very good’, ‘good’, ‘neither good nor bad’, ‘bad’, ‘very bad’;

3 between brackets the estimated proportion after further adjustment for parental educational level if this differed ≥3 percent points from the unadjusted proportion.

#### Personal variables of physical activity

Overall, very few children reported unfavourable attitudes, unfavourable preferences or low perceived behavioural control for doing physical activity. However, the majority of the children reported incorrect knowledge about the recommendation (Table 5). No clear differences across countries were observed, except that Spanish children more often reported low automaticity for participation in physical activity.

#### Family-environmental variables of sports participation

Overall, very few children reported unfavourable parental norms, low parental active encouragement, low parental support, low general and specific allowance. However, most children reported low parental co-participation (Table 5). Low parental modelling, not having family rules and low parental co-participation varied substantially between countries, but no clear pattern was observed.

#### Friend-environmental variables of sports participation

Overall, few children reported unfavourable friend norms and friend modelling, which was consistent across the countries.

### Television viewing

The descriptive results regarding the variables related to television viewing and between-country comparisons are presented in Table 6. We solely present the observed unadjusted values, because adjustment for age and sex did not substantially influence the estimates (i.e. change ≤2 percent points). Further adjustment for parental education only affected some estimates in the Dutch, Norwegian and Hungarian samples (≥3 percent points change). These adjusted estimates are presented between brackets in Table 5.

**Table 6:** Observed proportions (%) and ranks^1^ for the variables related to **TV watching** for the total sample and by country.

			Belgium	Greece	Hungary	Netherlands	Norway	Slovenia	Spain	Switzerland		Total
Question or statement (label or construct)	Answer categories^2^		n (%)	n (%)	n (%)	n (%)	n (%)	n (%)	n (%)	n (%)	n (%)	
**Personal**												
I think that watching television is…(attitude)	*Good; very good*	%	36	37	41	26	32	43	32	37	36	
		rank	2, 3	3	4	1	2	4	2	2, 3		
I think it is recommended for children my age to watch… (knowledge)	*more than 2 hours/day; don’t know*	%	19	16	25	**30**	26	15	11	16	20	
		rank	3	2, 3	4	5	4	2	1	2, 3		
I think watching too much television will make me fat (health belief)	*disagree a bit; fully disagree*	%	**41**	22	18	39	28	35	31	35	31	
		rank	5	1	1	4, 5	2	3	2, 3	3, 4		
I like watching television (preference)	*agree a bit; fully agree*	%	**84**	**54**	72	**83**	77	74	78	**58**	73	
		rank	5	1	2	4, 5	3	3	4	1		
Watching television is something that I do without even really thinking about (automaticity)	*agree a bit; fully agree*	%	**60**	**32**	43 (40) 3	**60**	54	45	**33**	36	45 (42) 3	
		rank	5	1	2	5	4	3	1	1, 2		
I find NOT watching television … (perceived behavioural control)	*Difficult; very difficult*	%	22	22	23	15	12	10	18	16	17	
		rank	5	5	5	2, 3	1, 2	1	4	3, 4		
**Family environment**												
If I watch television, my parents/care givers think this is… (parental norm)	*Good; - very good*	%	75	47	68	82 (78)^3^	64	59	59	63	64	
		rank	4	1	3	5	2, 3	2	2	2, 3		
How often do your parents/care givers watch television? (parental modelling)	*Always; often*	%	46	54	62	40 (37)^3^	33	45	36	**33**	44	
		rank	4	5	5	3	1	4	2, 3	1, 2		
Do you have a television in your bedroom? (availability)	*Yes*	%	**28 (23)** 3	44	**66**	44 (38)^3^	40 (37)^3^	**28** (23)^3^	**28**	**16**	38 (34)^3^	
		rank	2	4	5	4	3	2	2	1		
Do your parents/care givers have rules about how many hours per day you are allowed to watch television? (rules)	*No*	%	55	33	51	**59 (52)** 3	53	41	31	49	46 (43)^3^	
		rank	4, 5	1	3	5	3, 4	2	1	3		
My parents/caregivers allow me to watch television, whenever I want (allowance)	*agree a bit; fully agree*	%	**43**	26	**46**	**47 (38)** 3	25	38	**22**	**12**	33 (30)^3^	
		rank	5	3	4, 5	5	2	4	2	1		
If I ask my parents/care givers to watch television, I can do so (accessibility)	*Often; always*	%	55	53	75	56 (45)^3^	55	51	**30**	29	52 (49)^3^	
		rank	2	2	3	2	2	2	1	1		
How often do you watch television with your parents care givers? (co-participation)	*Every day or more often*	%	**45**	26	34	36	**12**	27	34	22	30	
		rank	6	2, 3	4, 5	5	1	3, 4	5	2		
**Friend environment**												
If I watch television my friends think this is (friend norm)	*good - very good*	%	96	78	**96**	94	**93**	**90**	94	**93**	92	
		Rank	4	1	3, 4	3, 4	2	2	3, 4	2, 3		
How often do your friends watch television? (friend modelling)	*often - always*	%	76	76	**89**	63 (60)^3^	**59**	75	74	**60**	72	
		rank	3	3	4	2	1	3	3	1, 2		

**Bold** – indicates that the observed proportion differs 10 or more percent points from the proportion in the Total sample.

1 rank orders are based on between-country comparisons by logistic regression and shown in the order of most favourable to least favourable. Countries with the same rank do not differ from each other (p>0.05).

2 potential categories are ‘fully disagree’, disagree a bit’, ‘neither disagree nor agree’, ‘agree a bit’, ‘fully agree’ or ‘always’, ‘often’, ‘sometimes’, ‘not often’, ‘never’ or ‘very good’, ‘good’, ‘neither good nor bad’, ‘bad’, ‘very bad’.

3 between brackets the estimated proportion after further adjustment for parental educational level if this differed ≥3 percent points from the unadjusted proportion.

#### Personal variables of television viewing

Overall, a substantial proportion of the children reported an unfavourable attitude, incorrect knowledge, unfavourable health belief or a low perceived behavioural control, which was consistent across the countries. Contrary, unfavourable preferences and high automaticity varied between countries. In general a more unfavourable pattern was observed in the Belgian and the Dutch sample, while a more favourable pattern was observed in the Greek and Swiss sample.

#### Family-environmental variables of television viewing

Most children reported unfavourable, non-restrictive family factors, but this varied substantially across countries for all variables (see Table 6). In general, Belgian, Hungarian and Dutch children most often reported unfavourable, non-restrictive family factors regarding television viewing, while this was less often observed in Spanish and Swiss children.

#### Friend-environmental variables of television viewing

Most children reported unfavourable friend norms and high modelling regarding television viewing. In general, Hungarian children most often reported unfavourable friend environmental variables.

## Discussion

The current study provides for the first time an overview of perceptions of schoolchildren in eight European countries regarding personal, family- and friend-environmental variables of specific energy balance-related behaviours (EBRB). In general, a majority of the surveyed children reported high preferences regarding the unhealthy behaviours (soft drink consumption and television viewing). Furthermore, only a few reported low preferences for the healthy EBRB (breakfast consumption and physical activity). However, knowledge regarding recommendations and health beliefs favouring the healthy behaviour were not often reported.

Our results showed that most children like the taste of soft drinks and that soft drinks are available in most homes. Taste preferences and home availability have been found to be strong correlates of soft drink intake among schoolchildren [18]–[20], [38], [39]. Therefore, it is a concern that soft drinks are perceived as highly available in most homes across Europe. We furthermore found that more than half of the children reported that there were no rules in place regarding soft drink intake and allowance to drink soft drinks and that accessibility to soft drinks was in general high. Previous studies have suggested that strict rules regarding soft drink intake is related to lower intake or a decrease in intake [15], [19], [21].

Besides these general trends, we observed large differences between countries in the children's perceptions towards soft drinks. Norwegian children appear to live in the least soft drink-friendly environment: Norwegian children least often reported that drinking soft drinks is good. They also experienced stricter parental norms and allowances and more favourable example behaviour of their parents and friends. Soft drink intakes are much lower in Norway as compared to most other countries included in the ENERGY project [2]. These healthy patterns might be the result of - or are at least supported by - the Norwegian health authority's goal to reduce the number of people consuming soft drinks and lemonade by 20% [40] leading to, among other things, structural and environmental changes, such as removal of vending machines in schools, and adapted guidelines related to marketing of unhealthy foods [41], [42]. This assumption is supported by a recently published study, reporting a decrease in sugar sweetened soft drink consumption in Norwegian children [42]. The observation that Norwegian children have a high liking for fizzy drinks despite their low intake levels is interesting. Sugary drinks is something that appeals to many children, because of a combination of causes, certainly including its sweet taste -that we have an innate preference for- and the carefully build image of many fizzy drinks. It may be that because in Norway availability and accessibility –and consumption- of such drinks is lower, that desire for such drinks is higher. The EnRG framework [29] indeed posits that individual preferences or attitudes may be less important as determinants of intake when availability is low; if fizzy drinks are just not available, it is just more difficult or even impossible to act upon your preferences, or these preferences may not have been developed.

The Dutch and Hungarian children appear to live in the most soft drink-friendly environments. This is in line with previously published results on soft drink intake levels, showing high intake levels in these countries [2].

Children generally reported positive perceptions and non-restrictive home environments regarding fruit juice. One explanation may be that most parents and children believe that fruit juice intake is healthy as it is widely marketed this way [43]. This belief has for a long time been supported by health recommendations that include so-called unsweetened fruit juices to increase daily fruit and vegetable intake [8]. Although the recommendation referred to unsweetened fruit juices, these fruit juices contain ‘natural’ sugar similar or exceeding sugar contents of regular soft drinks. Future health promotion efforts should help parents and children to put fruit juice intake in the right perspective, i.e. explain that 100% fruit juice is healthy, but also contains high amounts of sugar –comparable to sugar sweetened soft drinks- and therefore consumption should be limited to 1 glass a day in order to prevent excessive weight gain.

Previous studies have shown that breakfast skipping is a risk behaviour for overweight and that skipping breakfast may incorrectly be perceived as an effective strategy for weight management [9]. Even though only a few of the participating children reported unfavourable attitudes and low perceived behavioural control for eating breakfast, many children believed that eating breakfast could make them fat. Earlier studies found that weight concerns among adolescents and not believing that breakfast consumption helps to keep a healthy weight predicted future breakfast skipping. This indicates that adolescents perceive breakfast skipping as an effective strategy to lose or control weight [9], [44], while skipping breakfast has been associated with overweight [5], [45] and has inversely been related to cognitive function [46] and school performance [47]. Earlier studies have shown that a favourable and supportive family environment is of great importance for breakfast consumption among schoolchildren [20], [48], [49]. Our results indicate that very few children perceived unfavourable parental norms and only few reported that their parents rarely ate breakfast. Having family rules regarding breakfast has been associated with more frequent breakfast consumption [49], but a majority of the children reported to have no family rules regarding breakfast. On the other hand, a minority reported low parental encouragement, low co-participation and low availability. Rules may not be necessary if it is a habit for children to have breakfast and they have a supportive home environment. However, about half of the Spanish children reported that having breakfast was not an automaticity for them, while they also most often reported incorrect health beliefs. Future interventions should target the family environment and encourage parents to act as good role models [49], [50], this may lead to habitual breakfast intake in their children. Furthermore, interventions should educate children about the beneficial effects of having breakfast daily, and that skipping breakfast is not helping for weight management.

Despite the fact that many children do not comply with the physical activity recommendations [10], our results suggest that only a few children have unfavourable preferences, which is encouraging. Furthermore, children seem confident that they can do physical activity 1 hour per day, which is promising as self-efficacy or perceived behavioural control has been reported as an important determinant for physical activity [51], [52] The current study shows that knowledge of the recommendation and weight-related health beliefs regarding physical activity was limited, therefore children and their parents need to become more aware of the recommendations and health benefits of physical activity [53]. Moreover, the current findings suggest that parents in general may take a more active role in encouraging physical activity among their children by setting the right example and doing physical activity with their children, which all have been positively related to physical activity levels in children [12], [20], [54]. Especially in this age group parental support and encouragement is an important enabling factor, that may be required before personal factors can elicit their effect.

In general, children reported low restriction and high allowance towards television viewing. Since recent research shows that parental restriction is related to lower screen time [55], future interventions should target parents. On the other hand, it may be that parents start setting rules if their children watch too much television, and therefore our cross-sectional observation of the low restriction does not reflect actual television viewing [2]. While it may also be that parents do not like to set restricting rules as they prefer watching together rather them limiting TV time of their children. Interventions should therefore address parents and children and make both aware of the potential unhealthy effects of high TV time. Furthermore, the questions only addressed TV viewing, which is just one major screen activity and children may have different believes or may experience different rules regarding other screen activities, such as computer gaming or tablet use.

Some between-country differences and patterns are noteworthy. The Belgium and Dutch children showed unfavourable patterns with respect to soft drink and fruit juice intake and TV viewing-related variables. Previous publications on these study populations already showed that the Dutch children had high levels of soft drink intake [2], which might thus be explained by those unfavourable patterns, but this does not hold for Belgium children. On the contrary, previous results indicated unfavourable patterns in EBRB among Greek children, while this pattern was not found for the personal and social environmental beliefs reported in in the present study, except regarding breakfast intake. Few Greek children have breakfast with their parents and few Greek parents eat breakfast. Our previously reported finding in the ENERGY cross sectional study [27] that breakfast skipping is a problem among Greek primary schoolchildren and the current observation of an unfavourable home environment is in line with a recent publication [56]. Therefore, in the Netherlands and Belgium special attention should be devoted to beliefs and home influences related to soft drink and fruit juice intake, while in Greece parental role modelling and rule setting regarding breakfast consumption should be emphasised.

The strength of the current study is the large sample size and the wide range of variables at the personal and social environmental levels assessed. Moreover, the large majority of the items showed good test-retest reliability [26]. However, a limitation is that we relied on self-report, which may have led to social desirability bias. Furthermore, based on social-cultural and personal norms and values, children may have interpreted possible answers such as ‘often’ and ‘always’ differently. Finally, relatively low construct validity was found which might indicate that children may have interpreted the questions differently than what the researchers originally intended. The relatively poor construct validity may also be the result of the method used to estimate construct validity and the absence of a golden standard for this. There is still no golden standard to estimate construct validity for cognitions towards health behaviours, and cognitive interviews may have evoked different interpretations of the questions and answers than the self-completed questions. Another possible limitation is that data collection in Switzerland took place somewhat later than in the other countries; however, seasonal differences in the potential correlates assessed seem unlikely. A potential limitation to the generalizability of the findings is the relatively low response rates at school level in some of the countries (the Netherlands, 5%; Belgium, 29% and Norway, 36%). School boards that already had a focus on health may have been more likely to agree on participation in the study, resulting in more favourable answers. A final important limitation is that the present study reported on patterns and country-differences across Europe in beliefs and perceptions, but did not investigate the associations of such beliefs and perceptions with the EBRB. Such associations have been studied before [11], [12], [15], [20], [54], [57]. Some of these associations have been reported for the ENERGY data before [35], [58]–[60]. Given the fact that our data are cross-sectional, such associations are difficult to interpret, because of the probable reciprocal relationships between such beliefs and the behaviours. Furthermore, adding results on associations with behaviours and/or health outcomes would have made the current study more complex. Therefore the current study only focussed on descriptive information, which is in itself very relevant for public health professionals.

## Conclusions

This study shows that the majority of the European children have favourable attitudes towards the healthy behaviours, but many children across Europe have personal beliefs and are exposed to social environments that may not be supportive to healthy health behaviour. Moreover, the large differences in personal and social environmental variables across Europe argue for implementing different strategies in the different European countries.
